# Competency-based assessment as a reliable skill building strategy for allied ophthalmic personnel

**Published:** 2018-07-31

**Authors:** Sunita Arora, Umang Mathur, Parul Datta

**Affiliations:** 1 Programme Manager: Dr. Shroff's Charity Eye Hospital, New Delhi, India.; 2 Executive Director: Dr. Shroff's Charity Eye Hospital, New Delhi, India.; 3 Associate Medical Director: Dr. Shroff's Charity Eye Hospital, New Delhi, India.

**Developing a cadre of allied ophthalmic personnel poses a particular challenge for ophthalmic institutes as there are no accredited standards or curricula in most of the countries in the developing world. Competency- based assessments are gaining acceptance as they allow students to demonstrate mastery over a subject and earn competency without adhering to a rigid course schedule.**

**Figure:**
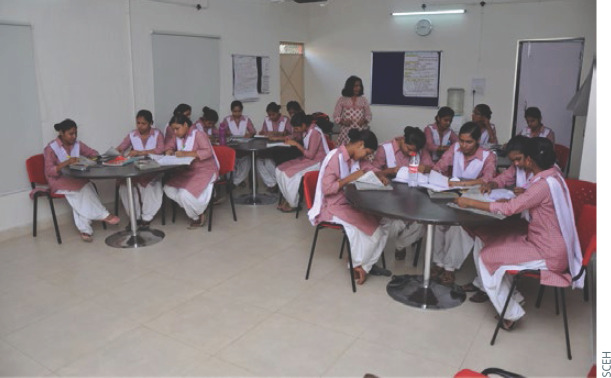
A teacher's role is to facilitate students to take a lead in their own learning. INDIA

Developing a cadre of allied ophthalmic personnel poses a particular challenge for ophthalmic institutes as there are no accredited standards or curricula in most of the countries in the developing world. In such a scenario, it becomes even more relevant to have a robust rubric for assessing skills and knowledge to ensure that this critical workforce gains a desirable level of competence.

A competency is the capability to apply or use a set of related knowledge, skills, and abilities required to successfully perform “critical work functions” or tasks in a defined work setting. Competency-based assessment (CBA) is a process that determines whether a person meets the standards of performance required for a job. It ensures greater accountability, flexibility, and it is learner-centric. See Table 1, for an example of CBA framework.

In particular, the model has garnered a lot of attention from policymakers and accreditation agencies. CBA allows students to demonstrate mastery over a subject and earn competency without adhering to a rigid course schedule. As soon as a student can prove mastery of a particular set of competencies, he or she is free to move on to the next level. Inclusion of CBA in curriculum of allied ophthalmic personnel has the potential for assuring:

- quality and extent of learning,
- shortening the course duration,
- developing stackable credentials that ease students' transition between learning and work, and
- reducing the overall cost of education

Considering the rising popularity of CBA, we at the Dr. Shroff's Charity Eye Hospital (SCEH) attempted to adapt CBA into our training and assessment. CBA is a formative approach to assessment compared to the traditional method which is restricted to giving ranks, marks and grades. The process we followed was:

- Revisiting and revising the curriculum of ophthalmic paramedics to align with existing standards
- Identifying core competencies of allied ophthalmic personnel and summarising those core capabilities that are important across all jobs that we believed contributed to the hospital's overall success
  - *Patient care*
  - *Medical knowledge*
  - *Professionalism, inter-personal and communication skills*
  - *Technical and scientific Skills*
  - *Community and health services*
- Identifying areas of assessment for each core competency:
  - *Mapping the areas of assessment into the various levels of performance such as novice, beginner, advanced beginner and competent and each level of competence is well-defined. For example, a novice observes an activity while the one who has mastered the competency can even supervise others.*
- Outline the tasks required to be performed for each area of competency:
  - *For example, for pupillary evaluation, one of the tasks can be, checks pupil for shape, size and reaction under varying illumination levels.*
- Scoring criteria can be laid down for each milestone for instance 2 for novice, 3 for the beginner, 4 for the advanced beginner and 5 for the competent.
- Students can be involved in creation of competency framework development so that they know what they are supposed to achieve to attain a specific level of competence.

**Table 1:** An example for CBA to assess visual acuity

Medical Knowledge					
Area	Activity	Novice (score = 2)	Beginner (score = 3)	Advanced beginner (score = 4)	Competent (score = 5)
**Visual acuity**	Ability to determine the visual acuity of the patient	Needs assistance to measure visual acuity appropriately and has limited knowledge	Measures visual acuities with Snellen charts	Measures visual acuities appropriately with correct usage of appropriate charts	Accurate measurement of visual acuities with respect to age with correct usage of appropriate charts
		Needs assistance in documentation of the findings	Measures monocular visual acuity and may measure binocular visual acuity for distance and near	Measures both monocular and binocular visual acuity for distance and near	Measures both monocular and binocular visual acuity for distance and near appropriately
			Assesses visual acuity with presenting glass prescription	Assesses visual acuity with presenting glass prescription	Assesses visual acuity with presenting glass prescription
			Assesses pinhole vision	Assesses pinhole vision when indicated	Assesses pinhole vision whenever indicated
			Documents the findings	Assesses visual acuity in special cases (Nystagmus nonverbal cases) appropriately	Assesses visual acuity in special cases (Nystagmus nonverbal cases) appropriately
				Documents the findings properly	Does proper documentation of the findings

A lot of effort is required in defining the areas of competence in a manner that remain unbiased, charts progressive development, and at the same time can demonstrate individual trajectory of competency acquisition. Subject matter experts review the competencies created, modify them and try to ensure that resulting competencies reflect all that a trainee must know and be able to do by the end of the course. We also field tested the competencies and continue to revise them to ensure that individualised learning is measured. Standard operating procedures were developed to ensure smooth transmission of training.

**Figure:**
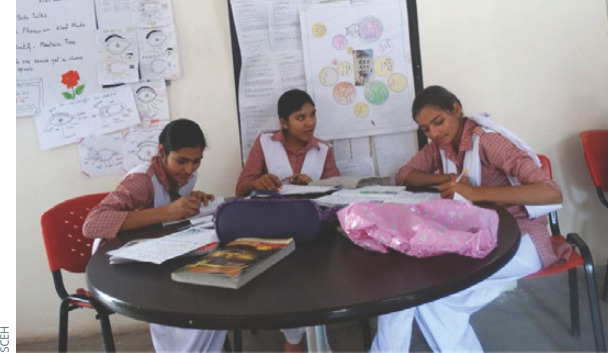
Assessments are done in multiple ways to get a holistic view of a student's understanding. INDIA

We realised that effective assessment is the driving force behind the conversion of the traditional system of teaching to a competency-based education programme. To have better results, the lessons were altered from lectures to supportive/remedial sessions that reach out to students who need additional help. The trainers were selected carefully and were trained to use the outlined competencies. They were counseled for their role as facilitators to help students to take a lead role in acquiring the outlined competencies in a stipulated time. Adoption of CBA was quite useful as students gained clarity about the level of competency they have achieved. It provided progression of growth and encouraged self-directed remediation.

## Potential downsides

On the flip side, CBA can give an impression of “expertise” (as opposed to achieving “competence”). At times students shy away from disclosing difficulties with an innate fear that they will not be pronounced competent. Students feel that they are solely responsible for carving their own learning progression while most of the time it is based on set training schedule. CBAs don't assess “soft skills” that are just as crucial to a successful professional. These could be skills like timeliness, tidiness or team work.

To address such limitations we measure each competency more than once, in multiple ways and by more than one person. In order to get a holistic view about a student, a battery of tests like multiple choice tests, question papers, presentations, log book, case studies, peer-review etc. can also be used to compare them with findings of CBA.

CBA provides an allied ophthalmic professional, transparent job expectations and a potent tool for performance assessment that provides an advancement path. CBA reduces subjectivity and creates a more positive work environment. Since it is new and more research is needed, we should be cautious in relying on results from a single method of assessment. We recommend that CBA be used in conjunction with other formative and summative assessment techniques to supplement the overall assessment of allied ophthalmic personnel rather than using it as standalone method of assessment.
